# Loss of *DIP2C* in RKO cells stimulates changes in DNA methylation and epithelial-mesenchymal transition

**DOI:** 10.1186/s12885-017-3472-5

**Published:** 2017-07-17

**Authors:** Chatarina Larsson, Muhammad Akhtar Ali, Tatjana Pandzic, Anders M. Lindroth, Liqun He, Tobias Sjöblom

**Affiliations:** 1 Department of Immunology, Genetics and Pathology, Uppsala University, Rudbeck Laboratory, Dag Hammarskjölds väg 20, SE-751 85 Uppsala, Sweden; 2 Current address: Department of Medical Biochemistry and Microbiology, Uppsala University, BMC, Husargatan 3, SE-751 23 Uppsala, Sweden; 3 Department of System Cancer Science, Graduate School of Cancer Science and Policy, National Cancer Center, 323 Ilsan-ro, Ilsandong-gu, 10408 Goyang-si, Republic of Korea; 4 Department of Neurosurgery, Tianjin Medical University General Hospital, Tianjin Neurological Institute, Key Laboratory of Post-Neuroinjury Neuro-Repair and Regeneration in Central Nervous System, Ministry of Education and Tianjin City, 300052 Tianjin, China

**Keywords:** Cancer, *DIP2C*, Gene knockout, rAAV-mediated gene targeting, Tumour cell biology, DNA methylation, Epithelial-mesenchymal transition (EMT)

## Abstract

**Background:**

The disco-interacting protein 2 homolog C (*DIP2C*) gene is an uncharacterized gene found mutated in a subset of breast and lung cancers. To understand the role of *DIP2C* in tumour development we studied the gene in human cancer cells.

**Methods:**

We engineered human *DIP2C* knockout cells by genome editing in cancer cells. The growth properties of the engineered cells were characterised and transcriptome and methylation analyses were carried out to identify pathways deregulated by inactivation of *DIP2C*. Effects on cell death pathways and epithelial-mesenchymal transition traits were studied based on the results from expression profiling.

**Results:**

Knockout of *DIP2C* in RKO cells resulted in cell enlargement and growth retardation. Expression profiling revealed 780 genes for which the expression level was affected by the loss of *DIP2C*, including the tumour-suppressor encoding *CDKN2A* gene, the epithelial-mesenchymal transition (EMT) regulator-encoding *ZEB1*, and *CD44* and *CD24* that encode breast cancer stem cell markers. Analysis of DNA methylation showed more than 30,000 sites affected by differential methylation, the majority of which were hypomethylated following loss of *DIP2C*. Changes in DNA methylation at promoter regions were strongly correlated to changes in gene expression, and genes involved with EMT and cell death were enriched among the differentially regulated genes. The *DIP2C* knockout cells had higher wound closing capacity and showed an increase in the proportion of cells positive for cellular senescence markers.

**Conclusions:**

Loss of *DIP2C* triggers substantial DNA methylation and gene expression changes, cellular senescence and epithelial-mesenchymal transition in cancer cells.

**Electronic supplementary material:**

The online version of this article (doi:10.1186/s12885-017-3472-5) contains supplementary material, which is available to authorized users.

## Background

The disco-interacting protein 2 homolog C (*DIP2C*), an uncharacterised gene expressed at high level in most human solid tissues and adult tumour types [1], was identified by us as a putative cancer gene in exome-wide mutational analyses of hormone-receptor negative breast tumours [2, 3]. Further studies have estimated the *DIP2C* somatic mutation prevalence at ~5% of breast cancer cases [4]. Recently, *DIP2C* was also found mutated in 9-14% of small-cell lung cancers [5], strengthening the evidence for a role in tumorigenesis.

Conserved across species, the human DIP2 family proteins DIP2A, DIP2B and DIP2C are highly similar, with DIP2C and DIP2B sharing 72.2% amino acid identity [6]. All three proteins are predicted to contain DMAP1 binding (pfam06464) and AMP binding (pfam00501) domains, which give properties of binding to the transcriptional co-repressor DNA methyltransferase 1 associated protein 1 (DMAP1), and acting enzymatically via an ATP-dependent covalent binding of AMP to their substrate, respectively. The most studied family member, DIP2A, is a potential cell membrane receptor for Follistatin-like 1 (FSTL1), a secreted protein with possible role in e.g. regulation of embryonic tissue formation, joint inflammation and allograft tolerance [7, 8]. Nervous-system specific expression of Dip2 protein has been shown in mouse and Drosophila during embryonic development [9], which is interesting considering that all three isoforms are associated with neurodevelopmental disorders. The *DIP2A* gene is a candidate for developmental dyslexia and autism [10, 11], DIP2B deficiency has been associated with mental retardation [6], and DIP2C has been implicated in developmental delay [12]. While *DIP2A* lacks known association to cancer development, an SNP associated with *DIP2B* expression has been proposed to affect colorectal cancer risk [13]. Thus far *DIP2C* is the only family member that has been identified as a candidate cancer gene through somatic mutation analysis.

Mutations found in breast cancers are predicted to inactivate DIP2C function [4]. To investigate the role of *DIP2C* inactivation in human cancer and identify processes affected by the activity of this gene we engineered and characterised human *DIP2C* knockout cell lines which revealed that loss of DIP2C affects cell growth, cell cycle regulation, and migratory capacity, potentially through regulation of DNA methylation.

## Methods

### Targeting construct

The *DIP2C* knockout construct was designed using CCDS7054.1. Primers are listed in Additional file 1: Tables S1 (PCR) and S2 (RT-qPCR). Exon 9 was chosen for deletion based on its location early in the transcript, as well as conforming to criteria for successful rAAV-mediated gene targeting as described in literature [14, 15]. Homology arm (HA) sequences were PCR amplified from RKO (ATCC, Manassas, VA, USA) gDNA using Platinum Taq DNA Polymerase High Fidelity (Invitrogen, Carlsbad, CA, USA) and a touchdown cycling protocol with restriction endonuclease-site tagged primers 1-4. The amplified HAs were then digested with the respective restriction endonucleases (Fermentas/Thermo Scientific, Waltham, MA, USA). The selection cassette, containing an IRES *neo* gene flanked by *LoxP* sites was excised from the pSEPT vector [16] by *Xba*I and *Xho*I (Fermentas) digestion. The AAV vector backbone with inverted terminal repeats (ITRs) and an ampicillin bacterial resistance marker was released from the pAAV-MCS vector (Stratagene, San Diego, CA, USA) by *Not*I digestion, and gel purified alongside the excised selection cassette and the digested HAs. The 5′ HA, selection cassette, and 3′ HA were ligated between the AAV vector backbone ITRs using T4 DNA ligase (Fermentas). Fragment cloning and orientation was confirmed by PCR (primers 5-8) and Sanger sequencing. The DIP2C-rAAV virus particles were produced by transfection of 70% confluent AAV-293 cells (Stratagene; cultured in DMEM, 10% FBS and 1% penicillin/streptomycin (PEST) (all from Gibco/Life Technologies, Carlsbad, CA, USA)) with Lipofectamine (Invitrogen) and 5 μg each of pAAV-RC, pHELPER (Stratagene) and the targeting construct, with harvesting of the cell lysate after 48 h as described [15].

### Cell lines and targeting

The human colorectal cancer cell line RKO (ATCC, CRL-2577) was cultured in McCoy’s 5A (Gibco), 10% FBS and 1% PEST. Human immortalized mammary epithelial cell line MCF10a (ATCC, CRL-10317) was cultured in DMEM-F12 (Gibco), 5% horse serum (Gibco), 0.02 μg/ml EGF (PeproTech, Rocky Hill, NJ, USA), 10 μg/ml Insulin (Sigma-Aldrich, St. Louis, MO, USA), 0.5 μg/ml Hydrocortisone (Sigma-Aldrich), 0.1 μg/ml Cholera Toxin (Sigma-Aldrich) and 1% PEST. Cells were transfected with DIP2C-rAAV as described [15], and selected for 2 weeks at limiting dilution with 0.8 mg/ml (RKO) or 0.1 mg/ml (MCF10a) Geneticin (Gibco). Single-cell clones with site-specific construct integration were identified by PCR (primer pairs 9 + 10, 6 + 9, 7 + 10). The *neo* selection cassette was removed by Ad-Cre virus (Vector Biolabs, Malvern, PA, USA) infection [15]. Single-cell clones identified by PCR (primers 11 + 12) to lack selection cassette were verified to be sensitive to Geneticin. A second targeting round was carried out as described above to generate homozygous knockouts. For overexpression, parental RKO cells were transfected with 2.5 μg Myc-DDK tagged DIP2C TrueORF Gold cDNA clone expression vector (RC209325, OriGene, Rockville, MD, USA) and Lipofectamine 2000 (Invitrogen) and enriched for stable integration in 0.8 mg/ml Geneticin. Single-cell clones overexpressing *DIP2C* were identified by RT-qPCR (primers DIP2C F and DIP2C R), and construct integration was verified in gDNA by PCR (primers 13-16). The RKO cells were authenticated by STR profiling at ATCC (June 2016). The *DIP2C* knockout and overexpression cells had 86-97% of their respective STR alleles in common with the parental RKO cells. The MSI status of RKO cells and establishment of clones from single cells are plausible sources for variation in alleles, as suggested by others [17]. Cells were tested for mycoplasma using the MycoAlert mycoplasma detection kit (Lonza, Basel, Switzerland).

### Cell morphology and growth

Cells were imaged with an IncuCyte HD (Essen BioScience, Ann Arbor, MI, USA) every 6-12 h during culturing, recording cell confluency for growth curves. Alternatively, since *DIP2C* KO cells differed in size, growth curves were generated by collection and counting of cells at set time points. For cell size comparison, cell diameter data was collected from the Cedex cell counter (Roche Innovatis, Switzerland) at eight occasions for a total of >5000 cells/cell line. For colony formation analyses, 400 cells plated in triplicate in 6-well plates were stained with 5% methylene blue in methanol after 10 days and colonies quantified. The plating efficiency was calculated as the number of obtained colonies divided by the number of seeded cells. For cell cycle analysis, equal numbers of cells fixed in ice cold 70% ethanol were stained with FxCycle PI/RNase staining solution (Molecular Probes, Eugene, OR, USA) for 15 min at room temperature, washed once in PBS, and analysed using a FlowSight flow cytometer (Amnis, Merck Millipore, Darmstadt, Germany).

### Western blot

Samples lysed in RIPA buffer (25 mM Tris-HCl, pH 7.6, 150 mM NaCl, 1% NP40, 0.1% SDS) with protease inhibitors (Roche) were separated on NuPAGE Novex 4-12% Bis-Tris protein gels with 1× Novex Bis-Tris MOPS running buffer (Life Technologies), transferred onto Hybond C-Extra membranes (Amersham Biosciences, UK), and probed with mouse anti-DIP2C antibody (SAB1411930, Sigma Aldrich) diluted 1:300, rabbit anti-ZEB1 (HPA027524, Atlas Antibodies, Stockholm Sweden) diluted 1:300, mouse anti-p53 DO-1 antibody (sc-126) diluted 1:1000, and rabbit anti-p21 H-164 antibody (sc-756) diluted 1:300 (both from Santa Cruz Biotechnology, Dallas, TX, USA). Secondary antibodies Pierce goat-anti-mouse (#31430) and goat-anti-rabbit (#31460) (Thermo Scientific) were diluted at 1:10,000. Mouse anti-β-actin (A5441, Sigma Aldrich) was used as loading control. Immunoreactive proteins were visualized using SuperSignal West Femto Maximum Sensitivity Substrate (Thermo Scientific) on the ImageQuant LAS 4000 imaging system (GE Healthcare) (exposure p53 – 10-20 s, p21 – 1-3 min, β-actin - 0.5-1 s). Relative protein amounts were quantified by densitometric analysis using ImageJ [18].

### RNA sequencing

Integrity and concentration of RNA was determined using a RNA 6000 nano chip on the Bioanalyzer 2100 instrument (Agilent, Santa Clara, CA, USA). Samples were sequenced on the Ion Proton system (Ion Torrent/Life Technologies) at the SciLife Lab NGI Uppsala platform. RNA-sequencing reads were aligned to the UCSC database hg19 human genome sequence (downloaded with the gene coordinate references via the Illumina iGenomes project [19]) using Tophat2 (version 2.0.4) [20]. Gene expression level quantification and identification of differentially expressed genes was done using Cufflinks (version 2.1.1) [21]. The ten most up- and downregulated genes across samples were selected for RT-qPCR validation, excluding genes without data in a *DIP2C* ^*−/−*^ clone, and genes with data in <2 knockout samples. The GSEA MSigDB v5.0 [22] Hallmarks gene set [23] and the DAVID Bioinformatics Resources [24, 25] (v6.8, annotation category GOTERM_BP_FAT) were used to compute overlaps and identify enriched biological functions for regulated genes. The entire RNAseq data set has been deposited in the NCBI Gene Expression Omnibus (GEO) database [26] (accession number GSE80746).

### RT-qPCR

Primers for qPCR were designed online using ProbeFinder (Roche), or retrieved from literature *(IL13RA2, p14* ^*ARF*^, *p16* ^*INK4a*^) [27, 28], and evaluated by a five-step 1:5 dilution standard curve. TATA-box binding protein (*TBP)* was selected as reference gene by stability evaluation across independent RKO and *DIP2C* knockout samples using the Cotton EST database RefFinder tool [29]. The Maxima H minus First strand cDNA synthesis kit (Thermo Scientific) with random primers was used for cDNA synthesis. Technical triplicate 20 μl qPCR reactions with 1× Maxima SYBR Green/ROX qPCR Master Mix (Thermo Scientific) and 0.3 μM of each primer were run on the StepOne Real-Time PCR system (Applied Biosystems, Foster City, CA, USA), including controls for gDNA contamination, and no-template controls. Data was analysed by the ΔΔCt method using the StepOne software v2.1 (Applied Biosystems).

### DNA methylation analysis

Genomic DNA was analysed on the Infinium HumanMethylation 450 K Bead Chip array (Illumina, San Diego, CA, USA) at the Uppsala SciLife Lab NGI SNP/SEQ technology platform. Raw data IDAT files were processed at the Uppsala Array and analysis facility. Color balance adjustment and background correction [30] was performed in the statistical computing language R [31], using the methylumi package from the Bioconductor project [32]. Filtering was performed using the “pfilter” function with default settings from the R-package watermelon, available from the Bioconductor project [32], removing sites with bead count <3 in >5% of the samples, and sites where >1% of samples had a detection *p* value >0.05. Quantile normalization of the pooled signal intensities of methylated and unmethylated probes was done before calculation of β-values with the “nanet” method from the same R-package. The probe type bias in the Illumina Infinium technology was eliminated by beta mixture quantile dilation (BMIQ), as suggested by others [33]. CpG sites were annotated to RefSeq genes according to the Human Methylation 450 k manifest file version 1.2, and selected according to gene context. The β-value median for sites in each RefSeq gene was calculated, using CpG sites annotated to more than one gene in the calculation of the median for all those genes, and used to calculate beta-diff values. Functional enrichment analysis was performed using the GSEA MSigDB Hallmarks gene set as described above. The DNA methylation data set was deposited in the NCBI GEO database [26] (accession number GSE86402).

### Senescence and scratch assays

Sub-confluent cells were stained using the Senescence β-Galactosidase Staining Kit (Cell Signaling Technology, Danvers, MA, USA), and blue staining was visualised by light microscopy. Results were quantified using the Cell counter plugin for ImageJ. Confluent cell monolayers in 6- or 12-well plates were scratched with a 200 μl pipet tip, washed with fresh medium at least three times and imaged by light microscopy or in an IncuCyte HD instrument. For microscopy images, the TScratch software [34] was used to calculate the open wound area at defined time points. For IncuCyte images, the surface covered by cells calculated by the instrument was used to determine the open wound area in accordance with TScratch.

## Results

### Generation of DIP2C knockout cells

**Fig. 1:**
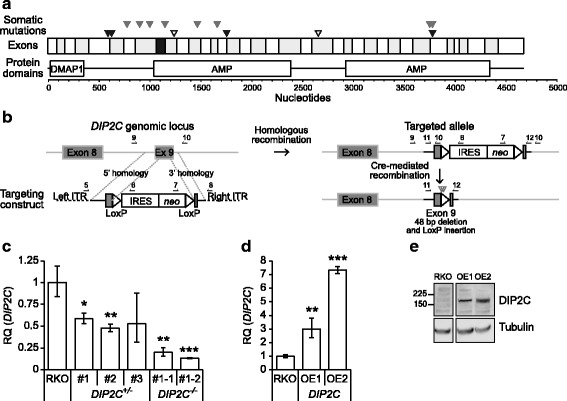
*DIP2C* knock-out by rAAV-mediated deletion of 48 bp in exon 9 in human cancer cells. **a** Coding sequence and predicted structural protein domains of *DIP2C*. Alternating exons are indicated and exon 9, targeted for deletion of 48 bp, shown in black. Triangles represent mutations found in cancer; black - breast cancer [4], grey - lung cancer [5], filled – missense mutation, open – frameshift mutation. Coding exons and domains from Ensembl ENST00000280886.11, UTRs not shown. **b** The targeting vector with regions homologous to the 5′ and 3′ ends of *DIP2C* exon 9 and surrounding intronic sequence includes a promoter trap selectable neo marker (IRES *neo*), *LoxP* sites (triangular) for selection cassette removal, and AAV inverted terminal repeat (ITR) sequences (dashed lines) mediating integration into the genome. Genomic *DIP2C* alleles are targeted through homologous recombination mediated by rAAV, as shown by dotted lines. Correct integrations are identified by PCR after enrichment by *neo* selection. Cre recombinase excises the selectable marker, leaving one *LoxP* sequence at the integration site. The targeted deletion leads to frameshift during translation. In addition STOP codons in the vector sequence ensure protein truncation (open grey triangles). Genomic locations of exons 8 and 9 are shown before and after targeting. Black lines – vector sequence, grey lines – genomic sequence. Primer sites are indicated by arrows. **c-d** Expression of *DIP2C* mRNA in *DIP2C* knockout (**c**) and overexpression (**d**) clones measured by RT-qPCR. Three biological replicates for parental RKO, two replicates for *DIP2C* ^*−/−*^ #1-1, and singleplex samples for the other clones were assayed in technical triplicates. Bars - relative quantity (RQ) minimum and maximum. **e** Western blot for DIP2C in *DIP2C* overexpression clones. Predicted MW 170.8 kDa. # - knockout clone, OE – overexpression clone, (*P < 0.05; **P < 0.01; ***P < 0.001; two-tailed Student’s t-test)

The *DIP2C* missense and frameshift mutations identified in breast cancer in previous studies [4] are located predominantly in the first half of the transcript but outside the predicted DMAP1 binding domain (Fig 1a). By recombinant adeno-associated virus (rAAV) mediated gene targeting we generated *DIP2C*-deficient human cells containing a genomic 48 bp deletion in *DIP2C* exon 9 (Fig. 1b). We first targeted *DIP2C* in the breast epithelial cell line MCF10a, but failed to identify construct integration despite several attempts (not shown). We then targeted the human colorectal cancer cell line RKO, also well known to function with rAAV technology, and obtained three heterozygous knock-out clones (*DIP2C* ^*+/−*^ #1-3) following screening of 605 Geneticin-resistant clones, indicating <1% targeting efficiency. Two homozygous RKO *DIP2C* knock-out clones (*DIP2C* ^*−/−*^ #1-1 and #1-2) were confirmed following additional rounds of targeting in *DIP2C* ^*+/−*^ #1. Approximately four-fold and two-fold reductions in *DIP2C* mRNA levels were shown in homozygous and heterozygous RKO-derived knock-out clones, respectively (Fig. 1c). To investigate the effects of DIP2C overexpression in the same cell system we selected for stable integration of Myc-DDK-tagged *DIP2C* in transfected parental RKO cells and established clones with 3-7 fold overexpression of *DIP2C* mRNA (Fig. 1d-e). The engineered isogenic cell system and the stable overexpression clones were then used to study the phenotypes associated with *DIP2C* expression in cancer cells.

### RNA expression analysis

**Fig. 2:**
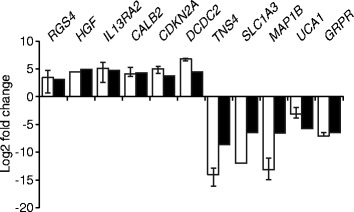
Loss of *DIP2C* induces changes in gene expression. Expression levels determined by RNA sequencing were verified by RT-qPCR for six upregulated and six downregulated genes in *DIP2C* ^*−/−*^ cells. White bars – RT-qPCR, black bars – RNA sequencing. Mean expression in two biological replicates of *DIP2C* ^*−/−*^ #1-1 and one *DIP2C* ^*−/−*^ #1-2 sample normalized to the mean of biological triplicates of parental RKO is shown for RT-qPCR data. All samples were run with technical triplicates. Error bars, SD. Mean log2 fold change for *DIP2C* ^*−/−*^ #1-1 and #1-2 relative parental RKO is shown for RNA sequencing data. *DCLK1* was not detectable by RT-qPCR in *DIP2C* ^*−/−*^ cells although stably expressed in parental RKO and is therefore not included in the graph

To investigate effects on gene expression levels we performed RNA sequencing, obtaining data for the expression of >4500 genes in each isogenic cell line. We identified 402 and 378 genes with more than 4-fold change up or down, respectively, in the *DIP2C* knockout clones under normal growth conditions (Additional file 2: Table S3). Strikingly, *DIP2C* ^*+/−*^ clones in many cases exhibited gene expression changes to the same magnitude as *DIP2C* ^*−/−*^ clones. From the gene lists, twelve of the most up-regulated (*RGS4*, *HGF*, *IL13RA2*, *CALB2*, *CDKN2A* and *DCDC2*) and down-regulated (*TNS4*, *SLC1A3*, *MAP1B*, *UCA1*, *GRPR* and *DCLK1*) genes were assayed by RT-qPCR in independent *DIP2C* ^*−/−*^ samples, validating gene expression changes consistent with the RNA sequencing data for all tested genes (Fig. 2). Gene set enrichment analysis (GSEA) [22] indicated function in epithelial to mesenchymal transition (EMT), apoptosis, inflammation and angiogenesis, along with genes regulated by different signalling pathways, such as TNF and KRAS, among the differentially expressed genes (Additional file 2: Table S4). Similarly, functional enrichment analysis using the DAVID bioinformatical resources [24] showed enrichment of genes involved with cell migration, blood vessel development and cell death (Additional file 2: Table S5). In summary, expression profiling revealed hundreds of genes, many of which implicated in processes linked to cancer, for which expression was altered by *DIP2C* knockout.

### Characterization of cell growth

**Fig. 3:**
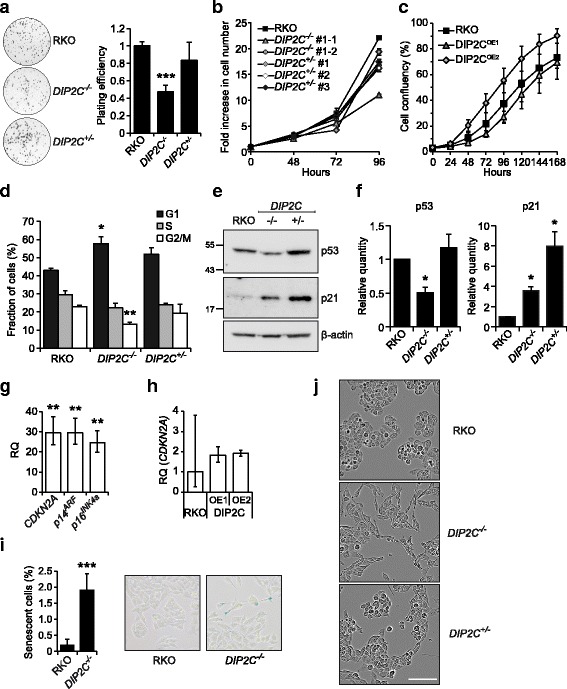
Loss of *DIP2C* affects RKO cell growth and cell cycle progression and induces senescence markers. **a** Colony formation assay for *DIP2C* knockout cells. Left, representative images of wells. Right, quantification of the mean plating efficiency of two independent experiments. All samples were normalized to the parental RKO used in each experiment. Error bars, SD. **b** Growth curve for *DIP2C* knockout cells. Cells seeded at equal density were harvested and counted once per day. Mean fold increase compared to the number of seeded cells and SD of technical duplicates is shown. The experiment was repeated at least twice for each clone. **c** Growth curve for RKO cells overexpressing wild type *DIP2C* showing mean cell confluency from daily imaging in an IncuCyte instrument. Error bars, SD of four replicate wells. **d** Cell cycle distribution determined by FACS. Error bars, SD of two independent experiments. **e** Western blot for p53 and p21 expression. Cell lines and targets as indicated. **f** Quantification of p53 and p21 immunoblots showing the mean of two independent experiments normalized to the β-actin loading control. Error bars, SD. **g-h** Quantification of expression from the *CDKN2A* locus by RT-qPCR with primers designed to target either multiple *CDKN2A* isoforms, or specifically *p14* ^*ARF*^ or *p16* ^*INK4a*^. Error bars - min and max relative quantity (RQ). **g** Mean of two biological *DIP2C* ^*−/−*^ #1-1 replicates and one *DIP2C* ^*−/−*^ #1-2 sample normalized to parental RKO. **h** Expression of all *CDKN2A* isoforms in *DIP2C* overexpressing (OE) cells. **i** Staining for β-galactosidase activity at pH 6. Quantification shows mean fraction of positive cells in six images per sample, at least 2000 cells were counted. Error bars -SD. Representative micrographs, blue – positive stain. **i** Morphology of live cells under normal growth conditions. Scale bar 100 μm. Results for *DIP2C* ^*−/−*^ #1-1 and *DIP2C* ^*+/−*^ #1 are displayed in figure unless otherwise indicated (*P < 0.05; **P < 0.01; ***P < 0.001; two-tailed Student’s t-test)

Analysis of the growth of DIP2C knockout cells showed an approximately 50% decreased ability to form colonies and a 35-50% reduced growth rate for *DIP2C* ^*−/−*^ cells, whereas heterozygous *DIP2C* ^*+/−*^ cells grew slightly slower but did not show any statistically significant reduction in colony number and formed macroscopically larger colonies compared to parental RKO (Fig. 3a-b). Overexpression of *DIP2C* did not affect the growth rate of RKO cells (Fig. 3c). Cell cycle analysis showed an increased proportion of *DIP2C* ^−/−^ cells in G1 phase (Fig. 3d), indicating a possible G1 arrest. The RNA-seq data indicated altered mRNA expression of several known cell cycle regulators, including p21/CDKN1A, which is a known transcriptional target of p53. For the TP53 gene, a minor transcriptional downregulation was observed by RNA-seq (Ave Log2 fold change −0.78 in *DIP2C* ^*−/−*^ cells), however as p53 protein levels are regulated not only through transcription [35] we analysed p53 protein levels further. Consistent with transcriptional data, immunoblot for p53 and p21 showed upregulation of p21 whereas the p53 level was slightly decreased in *DIP2C* ^*−/−*^ cells compared to the parental RKO cells (Fig. 3e-f). Interestingly, the p53 level was unaltered in *DIP2C* ^*+/−*^ cells compared to parental RKO whereas the p21 level was upregulated almost 2-fold more in *DIP2C* ^*+/−*^ cells compared to *DIP2C* ^*−/−*^ cells. These results were consistent over repeated independent experiments. These results suggest that DIP2C activity affects cell growth by modulation of cell cycle regulation in the G1 phase through a p53-independent mechanism.

The cyclin dependent kinase inhibitor 2A (*CDKN2A*) locus encodes at least three related genes that serve as tumour suppressors, of which *p14* ^*ARF*^ and *p16* ^*INK4a*^ *,* which have distinct first exons but share exons two and three translated in different reading frames, are known cell cycle regulators with multiple links to human cancer [36]. To discern if the upregulation of *CDKN2A* observed by RNA sequencing was due to isoform-specific expression from the *CDKN2A* locus, we performed qPCR and found *p14* ^*ARF*^ and *p16* ^*INK4a*^ upregulated to the same extent in *DIP2C* ^*−/−*^ cells (Fig. 3g). Overexpression of *DIP2C* in RKO cells did not lead to changes in *CDKN2A* expression (Fig. 3h). As *p16* ^*INK4a*^ is a marker of cellular senescence we then stained cells for β-galactosidase activity at pH 6, which is another characteristic of senescent cells, detecting staining of up to 1.9% of *DIP2C* ^*−/−*^ cells, compared to 0.2% of parental RKO cells (Fig. 3i). Importantly, complete growth cessation was not detected for any engineered cell line during culturing for more than 2 months (not shown), suggesting that cellular senescence is a non-permanent state in these cells, or that only a subpopulation of cells express the markers. Cell enlargement is another sign of senescence and imaging of live cells revealed that *DIP2C* knock-out cells appeared stretched-out relative to parental RKO cells (Fig. 3j). Further examination of cell size in suspension revealed a 15.9% (SD = 8.9) increase in cell volume for knockout clones (*P* = 0.003, two-tailed Student’s t-test). Also *DIP2C* ^*+/−*^ cells were affected, displaying an intermediate morphological phenotype in relation to parental RKO and *DIP2C* ^*−/−*^ cells. In summary, these data show that altered levels of DIP2C triggers a senescence response in human RKO CRC cells.

### Analysis of cell migration and EMT markers

**Fig. 4:**
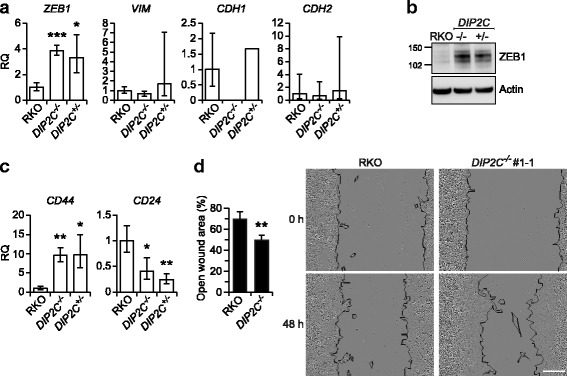
DIP2C knockout enhances cell motility and alters expression of EMT and cancer stem cell markers. **a** Quantification of the EMT regulator *ZEB1*, and EMT markers *VIM*, *CDH1* and *CDH2* by RT-qPCR. Mean expression for *DIP2C* ^*−/−*^ #1-1, and #1-2 and *DIP2C* ^*+/−*^ #1 and #2 is shown respectively. Error bars - min and max relative quantity (RQ). **b** Western blot for ZEB1. Predicted molecular weight 124 kDa. **c** Quantification of *CD44* and *CD24*, markers for breast cancer stem-like cell properties, by RT-qPCR*.* Mean expression for *DIP2C* ^*−/−*^ #1-1, and #1-2 and *DIP2C* ^*+/−*^ #1 and #2 is shown respectively. Error bars - min and max relative quantity (RQ). **d** Scratch assay showing the wound closing ability for *DIP2C* ^*−/−*^ #1-1. The open wound area was determined by comparison of cell confluency at time points 0 and 48 h after scratching of the surface. Error bars SD. RKO *n* = 5 images, *DIP2C* ^*−/−*^ *n* = 4 images. Representative micrographs are shown for both time points, cell borders were traced manually to enhance visualization and were not part of the analysis. The experiment was repeated three times. (*P < 0.05; **P < 0.01; ***P < 0.001; two-tailed Student’s t-test)

Functions associated with migratory capacity were enriched among the differentially expressed genes in *DIP2C* knockout cells. To investigate association to EMT, a process believed to influence metastasis by modulating cell motility [37, 38], we used RT-qPCR to analyze expression of Zinc finger E-box binding homeobox 1 (*ZEB1*) and Vimentin (*VIM*), which both were found ~4-fold upregulated by RNA sequencing. We validated transcriptional upregulation of the EMT regulator *ZEB1* in *DIP2C* ^*−/−*^ cells but could not validate upregulation of the EMT marker *VIM* (Fig 4a). We also investigated the frequently used EMT markers E-cadherin/cadherin 1 (*CDH1*) and N-cadherin/cadherin 2 (*CDH2*), detecting low or no expression in all cell clones, consistent with not being detected as expressed by RNA sequencing as well as previous reports of *CDH1* not being expressed in RKO cells [39]. Immunoblotting further validated the upregulation of ZEB1 in DIP2C KO clones (Fig. 4b). Epithelial-mesenchymal transition is associated with *CD44* high/*CD24* low*-*expressing breast cancer stem and stem-like cells [37, 38], and *CD44* was part of the GSEA Hallmarks EMT gene set found enriched among the differentially expressed genes in *DIP2C* ^*−/−*^ cells. By RT-qPCR we detected *CD44* and *CD24* to be more than 9-fold up- and 2.5-fold downregulated, respectively, in *DIP2C* ^*−/−*^ cells, validating the RNA sequencing data for these genes (Fig. 4c). We then functionally assessed the cells ability for migration by a scratch assay and observed a 30% higher capacity to close wounds for *DIP2C* ^*−/−*^ cells (Fig. 4d). This demonstrates that DIP2C activity affects traits and gene expression associated with transition to a more mesenchymal state in RKO cells.

### DNA methylation analysis

**Fig. 5:**
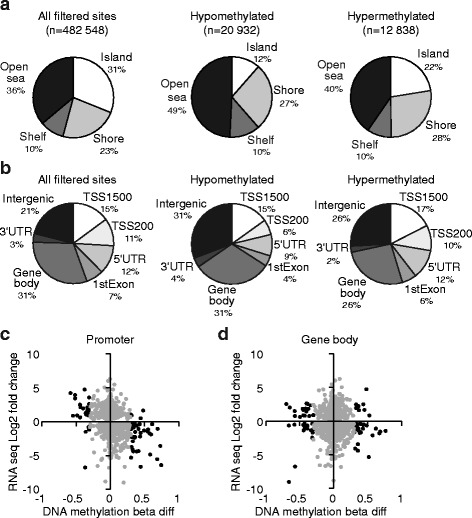
Promoter DNA methylation alteration is inversely correlated to gene expression changes in *DIP2C* ^*−/−*^ edited cells. **a** Distribution of assayed sites in relation to CpG islands. Shore - 0–2 kb from CpG island, Shelf - 2–4 kb from CpG island, Open sea - isolated CpGs in the genome. **b** Distribution of assayed sites in relation to gene context. TSS1500 – 1500 bp upstream the transcription start site (TSS), TSS200 – 200 bp upstream the TSS, UTR – untranslated region, 1st exon –exon 1 of gene transcript. In (**a**) and (**b**) the distribution of all sites with methylation data recorded in the assay is given as reference to the distribution of the differentially methylated sites (hypomethylated and hypermethylated sites with beta diff ≥│0.3│). The fraction of sites relative to the respective total number of sites is indicated for each category. **c-d** Plots of the change in DNA methylation (beta diff) at promoter regions (**c**) and gene bodies (**d**) and change in expression for the respective gene. Black – genes with beta diff ≥│0.3│ (promoter *n* = 72, gene body *n* = 74); grey – genes with beta diff <│0.3│. Promoter region includes TSS1500, TSS200, 5′ UTR and 1st exon

The DIP2 family proteins contain an N-terminal binding domain for DMAP1, a protein that participates in global maintenance DNA methylation by interaction with DNA methyltransferase 1 (DNMT1) [40, 41], and a SNP in *DIP2B* recently provided association to the DNA methylation process by being linked to DNA methylation variation in HapMap cell lines [42]. We therefore investigated DNA methylation in the *DIP2C* knockouts by array hybridization, obtaining beta values reflecting the methylation level in the range of 0-1 (representing 0-100% methylation) at 482548 genomic cytosine positions. We identified 33,700 differentially methylated CpG sites, of which 62% were hypomethylated (methylation level in KO cells was decreased ≥0.3 units), and 38% were hypermethylated (methylation level in KO cells was increased ≥0.3 units). While hypomethylated sites were more common in isolated CpGs across the genome (49% of hypo- and 40% of hypermethylated sites), and in intergenic genomic regions (31% hypo and 26% hyper), hypermethylation events were particularly elevated in CpG islands (22% hyper and 12% hypo), and more frequently associated with proximal promoter regions (45% hyper and 34% hypo) (Fig. 5a-b). Calculation of median methylation levels within the proximal promoter regions and bodies of genes revealed differential methylation of promoter regions in 520 genes and of gene bodies in 580 genes (Additional file 2: Tables S6-S7). Investigating the correlation between DNA methylation and transcript abundance, we observed a weak negative correlation between DNA methylation change at promoter regions and change in RNA expression level, which grew to strong (Pearson’s *r* = −0.66, *P* = 2.196 × 10^−10^) when considering only those genes that exhibited differential DNA methylation (Fig. 5c-d, Additional file 1: Table S8). Conversely, no correlation to gene expression level was observed for changes in gene body methylation. With the exception of one gene (*GRPR*), all the 28 genes with differential promoter methylation and ≥4-fold differential expression showed correlation between DNA methylation and gene expression changes in *DIP2C* ^*−/−*^ cells (Additional file 1: Table S9). Gene set enrichment analysis for the genes with differential promoter methylation revealed association with immune response function such as inflammation and coagulation, EMT and apoptosis (Additional file 2: Table S10). In summary these results show that there is correlation between altered promoter DNA methylation and differential gene expression in *DIP2C* knockout cells. Further, there are similarities in functions enriched among differentially methylated genes and genes exhibiting altered expression which support the hypothesis that *DIP2C* knockout alters gene expression partly through affected DNA methylation patterns.

## Discussion

Genomic profiling has revealed a large subset of genes as likely drivers of breast tumorigenesis, including the hitherto non-characterized *DIP2C* gene [2–4] which is interesting in association to cancer development since it may belong to the growing number of epigenetic regulators implicated in cancer. Here we exploited rAAV-mediated gene targeting to knock out one or two alleles of *DIP2C* in human cancer cells, enabling *DIP2C* to be studied under control of its endogenous promoter. Gene editing was attempted in two cell lines well established for use with the rAAV-targeting method, with the low targeting efficiency (no viable clones for untransformed mammary MCF10a cells and <1% targeted RKO cells) potentially owing to the significant changes in growth and transcription depending on loss of *DIP2C* that were observed in targeted RKO cells. This could mean that inactivating *DIP2C* mutations are dependent on additional genetic perturbation for cells to be able to survive their introduction. In support of this theory, *DIP2C* mutation was reported as a late event when the timing of mutations and chromosome rearrangements were investigated in a breast cancer genome [43]. If this is a general observation in affected tumours has however not been studied.

Even though the *DIP2C* mRNA level remained at ~50% of that of parental RKO, heterozygous *DIP2C* ^*+/−*^ cells also to some degree adopted the stretched morphology and decreased growth rate seen in *DIP2C* ^*−/−*^ cells, which suggests possible haploinsufficiency or a dominant negative effect of the damaged allele. The potentially functional DMAP1 binding domain spans amino acids 9-119 of DIP2C, encompassing sequence from exons 1-4. In the knockout cells a large part of DIP2C exon 9 was deleted, possibly generating a truncated protein with a preserved DMAP1 binding domain which could cause binding of non-functional DIP2C and blocking of normal DIP2C function in heterozygous clones. The predicted inactivating missense and frameshift mutations in breast cancer that motivated this study and the mutations later identified in lung cancer are all heterozygous and localized downstream of the DMAP1 binding domain [4] (Fig. 1a), suggesting that such a mechanism could be active also in patient tumours. Furthermore this observation is interesting as decreased expression levels of both *DIP2C* and *DIP2B* are associated with mental retardation [6, 12].

Disruption of DNA methylation patterns is a hallmark of cancer, and both promoter hypermethylation and global loss of DNA methylation is observed in cancers [44]. In *DIP2C* knockout cells hypermethylation was the dominating effect at CpG islands and in sites located closely upstream of transcription start sites, which agrees with the DNA methylation pattern associated with gene silencing [44, 45]. Typically heavily methylated in normal tissue [44], isolated CpGs in the genome were instead preferentially hypomethylated. Differential gene promoter methylation was shown correlated to changes in gene expression, particularly for those genes with more than fourfold differential expression, suggesting that *DIP2C* KO methylation defects directly influence gene expression. In contrast, as expected [44, 45], gene expression was not correlated to differential methylation at gene bodies. Although a physical interaction has not been demonstrated between DIP2C and DMAP1, for which DIP2C has a putative binding site, based on these results we cannot rule out that DIP2C is involved in the regulation of DNA methylation through this pathway or by another mechanism. Although important in cancer development [46], DNMT regulation during tumorigenesis is poorly understood. Both DNMT1 and DMAP1 have multiple known interaction partners [41, 47, 48], and DMAP1 has suggested roles not only in DNA methylation but also in histone acetylation and DNA repair [47, 49], suggesting areas of investigation for future studies of DIP2C function.

In *DIP2C* knockout cells, 10-15% of the functionally assigned differentially expressed genes were involved with regulation of cell death processes (Additional file 2: Table S5). The *DIP2C* knockout cells did not show signs of apoptosis but displayed multiple markers for cellular senescence, a mechanism activated in ageing cells or by different forms of cellular stress, such as oncogenic signalling, as protection against inappropriate growth signals [50]. Seemingly contradictory, senescent cells can secrete factors that e.g. promote EMT and inflammation, which could stimulate tumorigenic processes [51]. Functional consequences of cellular senescence induction in *DIP2C* ^*−/−*^ cells cannot be determined from this cell system, but interestingly EMT and inflammation were among processes enriched for in the differentially expressed and/or methylated genes. Notably, *DIP2C* is among seven genes on chromosome 10p14-15 whose loss has been associated with the ability to escape from senescence in cervical cancer [52]. Such ability is suggested to be an important mechanism in the progression from pre-malignant to malignant cells [50]. Overexpression of *DIP2C* in CRC cells did not induce senescence markers in the present study, which is consistent with literature on overexpression in primary human fibroblasts and keratinocytes [52].

Epithelial-mesenchymal transition is a reversible spectrum of transitory cell states where cells express different levels of epithelial and mesenchymal markers [37, 53]. The RKO cell line has increased mesenchymal characteristics compared to several other colorectal cancer cell lines, with low expression of cytoskeletal structure and cell adhesion proteins and high migration and invasive capability [54]. The EMT inducer ZEB1 is previously reported expressed in RKO cells [39], meaning that loss of *DIP2C* may augment the RKO EMT phenotype by further ZEB1 upregulation as suggested by the data presented herein. Furthermore, high *CD44* and low *CD24* expression, characteristics associated with the breast cancer stem cell phenotype and the EMT state, was revealed in *DIP2C* KO cells, with potential implications for treatment and metastasis ability [37, 38]. The epithelial and mesenchymal states impact the stages of tumorigenesis differently [37, 53], suggesting that timing may influence the effect of *DIP2C* mutations on tumour development. Here, DIP2C KO caused increased migration in the scratch assay, suggesting possible impact on e.g. the ability to metastasize.

## Conclusions

Functional studies of the genes that are altered in cancer will increase the understanding of the changes that are induced as normal cells transform into cancer cells. In this project significant phenotypic and transcriptional alterations were induced by loss of the candidate breast and lung cancer gene *DIP2C* in cancer cells. Transcriptional changes were correlated to altered DNA methylation, suggesting that DIP2C activity has a role in regulation of this process. Results from functional assays indicate that inactivating *DIP2C* mutations may function to promote metastasis through EMT induction, but *DIP2C* knockout also triggered a senescence response in the cells that could either stimulate or inhibit tumorigenesis depending on the context. The function of DIP2C in normal cells still remains to be determined, but based on these results we cannot rule out an association to DNA methylation processes through the predicted N-terminal DMAP1 binding domain.

## Additional files

Additional file 1: Table S1. - Primers for generation and validation of isogenic *DIP2C* cell lines and *DIP2C* overexpressing cells. Primers are indicated by their number in the text. Primers were purchased from Sigma Aldrich. **Table S2.** - Primers for RT-qPCR. Primer pairs for amplification of the transcript indicated by the respective name. Primers were purchased from Sigma Aldrich. **Table S8.** - Summary of DNA methylation analysis in *DIP2C* ^*−/−*^ cells. **Table S9.** - Genes with change in methylation (beta diff ≥│0.3│) at promoter sites and gene expression (log2 fold change ≥│2│) in *DIP2C* ^*−/−*^ #1-1 compared to RKO. (PDF 199 kb)

Additional file 2: Table S3. - Genes with ≥4 fold change in expression in DIP2C knockout cells. **Table S4.** - Gene set overlap results for differentially expressed genes (≥4-fold change up or down) investigated with GSEA MSigDB Hallmarks gene set. **Table S5.** - Functional annotation chart for differentially expressed genes (>4-fold change up or down) investigated with the DAVID Functional annotation tool using the GO Biological process (GO_BP_FAT) annotation category. **Table S6.** - Genes with ≥│0.3│ units change in median promoter DNA methylation in DIP2C knockout cells. **Table S7.** - Genes with ≥│0.3│ units change in median gene body DNA methylation in DIP2C knockout cells. **Table S10.** - Gene set overlap results for promoter differentially methylated genes (≥│0.3│ change in methylation level at promoter sites in DIP2C−/− #1-1) investigated with GSEA MSigDB Hallmarks gene set. (XLSX 152 kb)

## Abbreviations

CDH1: Cadherin 1
CDH2: Cadherin 2
CDKN2A: Cyclin dependent kinase inhibitor 2A
DIP2C: Disco-interacting protein 2 homolog C
DMAP1: DNA methyltransferase 1 associated protein 1
DNMT1: DNA methyltransferase 1, EMT, epithelial-mesenchymal transition
GSEA: Gene set enrichment analysis
HA: Homology arm
ITR: Inverted terminal repeat
rAAV: Recombinant adeno-associated virus
VIM: Vimentin
ZEB1: Zinc finger E-box binding homeobox 1
