# Supplementary material for: Loss of DIP2C in RKO cells stimulates changes in DNA methylation and epithelial-mesenchymal transition
Source: BMC Cancer. 2017 Jul 17;17:487. doi: 10.1186/s12885-017-3472-5 (PMC5513093; doi:10.1186/s12885-017-3472-5)
Supplement: Supplementary file 1 — - Primers for generation and validation of isogenic DIP2C cell lines and DIP2C overexpressing cells. Primers are indicated by their number in the text. Primers were purchased from Sigma Aldrich. Table S2. - Primers for RT-qPCR. Primer pairs for amplification of the transcript indicated by the respective name. Primers were purchased from Sigma Aldrich. Table S8. - Summary of DNA methylation analysis in DIP2C −/− cells. Table S9. - Genes with change in methylation (beta diff ≥│0.3│) at promoter sites and gene expression (log2 fold change ≥│2│) in DIP2C −/− #1-1 compared to RKO. (PDF 199 kb) [file 12885_2017_3472_MOESM1_ESM.pdf]

ADDITIONAL FILE 2

Loss of *DIP2C* in RKO Cells Stimulates Changes in DNA Methylation and Epithelial-Mesenchymal Transition

Chatarina Larsson, Muhammad Akhtar Ali, Tatjana Pandzic, Anders M. Lindroth, Liqun He, and Tobias Sjöblom

**Supplementary Table 1**	Primers for generation and validation of isogenic *DIP2C* cell lines and *DIP2C* overexpressing cells. Primers are indicated by their number in the text. Primers were purchased from Sigma Aldrich.
**Supplementary Table 2**	Primers for RT-qPCR. Primer pairs for amplification of the transcript indicated by the respective name. Primers were purchased from Sigma Aldrich.
**Supplementary Table 8**	Summary of DNA methylation analysis in *DIP2C*$^{-/-}$ cells.
**Supplementary Table 9**	Genes with change in methylation (beta diff≥ ∣0.3∣) at promoter sites and gene expression (log2 fold change ≥ ∣2∣) in *DIP2C*$^{-/-}$ #1-1 compared to RKO.

Supplementary Table 1. Primers for generation and validation of isogenic *DIP2C* cell lines and *DIP2C* overexpressing cells. Primers are indicated by their number in the text. Primers were purchased from Sigma Aldrich.

Primer no.	Name	Sequence (5' – 3')
1	P1:HA1F not1	TCAGGAGCGGCCGCCTTGACTACTGAGACGCTCTGG
2	P2:HA1R xba1(scr)	TCAGGATCTAGATGTGTAACTTACCAGGTAGAGTTGCGACCTTCATACTTCTTGTCC
3	P3:HA2F xho1	TCAGGACTCGAGCCTGGAGATAGGGTAAGTGC
4	P4:HA2R not1	TCAGGAGCGGCCGCAGAGGCCAAACGTGTTCAAT
5	AAV-Neo F	GCCTTTTGCTCACATGTCCT
6	AAV-Neo R	AGGGAGTACTCACCCCAACA
7	Neo-AAV F	TCGCCTTCTTGACGAGTTCT
8	Neo-AAV R	TGACGTATGCGGTGTGAAAT
9	Screening F	AAACAGTGATGGTACTGAAATAGAAA
10	Screening R	TGTGTAACTTACCAGGTAGCAGTT
11	Cre scr F	CATGATACGCAGGAAGCAGA
12	Cre scr R	GGGGGCATCTAAAGCACATA
13	ORF 5' scr F	GATCCGGTACCGAGGAGAT
14	ORF 5' scr R	TGAACACATGATGGCTCCAG
15	ORF 3' scr F	AGATGGATGGCCTCATGGT
16	ORF 3' scr R	TTGCTGCCAGATCCTCTTCT

Supplementary Table 2. Primers for RT-qPCR. Primer pairs for amplification of the transcript indicated by the respective name. Primers were purchased from Sigma Aldrich.

Primer name	Sequence	Used for
DIP2C F	TGCCTAAGCCTCGACAGAAG	DIP2C quantification
DIP2C R	TTCCCAGAGACCAGGTTCC	DIP2C quantification
TBP F	CGGCTGTTTAACTTCGCTTC	Reference gene
TBP R	CACACGCCAAGAAACAGTGA	Reference gene
CALB2 F	AAAAACTCAGATGGGAAAATCG	RNA seq validation
CALB2 R	TCTCTTCGGTTGGCAGGA	RNA seq validation
CDKN2A F	GTGGACCTGGCTGAGGAG	RNA seq validation
CDKN2A R	CTTTCAATCGGGGATGTCTG	RNA seq validation
DCDC2 F	GAATTCACAAGAAACCATTCCAA	RNA seq validation
DCDC2 R	CCCCGTGTTTCAGACCTC	RNA seq validation
DCLK1 F	AAGTCTGCAGCTCGATGGAT	RNA seq validation
DCLK1 R	AGCCTTCCTCCGACACTTCT	RNA seq validation
GRPR F	CCCGTGGAAGGGAATATACA	RNA seq validation
GRPR R	GCGGTACAGGTAGATGACATGAT	RNA seq validation
HGF F	CAGCATGTCCTCCTGCATC	RNA seq validation
HGF R	TCTTTTCCTTTGTCCCTCTGC	RNA seq validation
IL13RA2 F	TCTTGGAAACCTGGCATAGGTG	RNA seq validation
IL13RA2 R	GCCTCCAAATAGGGAAATCTGC	RNA seq validation
MAP1B F	GACGCTTTGTTGGAAGGAAA	RNA seq validation
MAP1B R	CTGAGTCATGAGTTGGGATCAG	RNA seq validation
RGS4 F	CGGCTTCTTGCTTGAGGA	RNA seq validation
RGS4 R	GGGAAGAATTGTGTTCACAGG	RNA seq validation
SLC1A3 F	AGCACTCATCACCGCTCTG	RNA seq validation
SLC1A3 R	GTCCACGCCATTGTTCTCTT	RNA seq validation
TNS4 F	GATGTCAGCTATATGTTTGGAAGC	RNA seq validation
TNS4 R	GGATCTGGAAGATGACTGATGG	RNA seq validation
UCA1 F	CCCAAGGAACATCTCACCAA	RNA seq validation
UCA1 R	GATGGTCCAAGGGGCTTC	RNA seq validation
P14 F	CCCTCGTGCTGATGCTACTG	RNA seq validation/senescence
P14 R	CATCATGACCTGGTCTTCTAGGAA	RNA seq validation/senescence
P16 F	GGGGGCACCAGAGGCAGT	RNA seq validation/senescence
P16 R	GGTTGTGGCGGGGGCAGTT	RNA seq validation/senescence
CD24 F	CACGCAGATTTATTCCAGTGAAAC	EMT gene set qPCR
CD24 R	GACCACGAAGAGACTGGCTGTT	EMT gene set qPCR
CD44 F	CCAGAAGGAACAGTGGTTTGGC	EMT gene set qPCR
CD44 R	ACTGTCCTCTGGGCTTGGTGTT	EMT gene set qPCR
CDH1 F	GCCTCCTGAAAAGAGAGTGGAAG	EMT gene set qPCR
CDH1 R	TGGCAGTGTCTCTCCAAATCCG	EMT gene set qPCR
CDH2 F	CCTCCAGAGTTTACTGCCATGAC	EMT gene set qPCR
CDH2 R	GTAGGATCTCCGCCACTGATTC	EMT gene set qPCR
VIM F	AGGCAAAGCAGGAGTCCACTGA	EMT gene set qPCR
VIM R	ATCTGGCGTTCCAGGGACTCAT	EMT gene set qPCR
ZEB1 F	TCAGTGCAGTCTTCTGAACCA	EMT gene set qPCR
ZEB1 R	GAGGCTGATCATTGTTCTTGG	EMT gene set qPCR

Supplementary Table 8. Summary of DNA methylation analysis in *DIP2C*$^{-/-}$ cells.

Ref gene group	Genes with DNA methylation data	Hypo-methylated[1]	Hyper-methylated[1]	Genes with DNA methylation and RNA seq data	Pearson's r all genes[2] (p value)	Pearson's r for genes with beta diff ≥│0.3│[3] (p value)
Gene body	19 545	385	194	2 316	-0.042 (p=0.0419)	-0.109 (p=0.356)
Promoter (TSS1500, TSS200, 5'UTR, 1st exon)	20 964	284	236	2 424	-0.278 ($p<2.2\times10^{-16}$)	-0.663 ($p=2.196\times10^{-10}$)

Data are based on *DIP2C*$^{-/-}$ #1-1.

[1]Hypomethylated genes - beta diff ≤-0.3; hypermethylated genes - beta diff ≥0.3.

[2]Pearson's product-moment correlation calculated in R describing correlation between change in DNA methylation and change in RNA expression for all genes with both DNA methylation and RNA sequencing data

[3]Pearson's product-moment correlation calculated in R describing correlation between change in DNA methylation and change in RNA expression for genes with beta diff ≥│0.3│

Supplementary table 9. Genes with change in methylation (beta diff≥|0.3|) at promoter sites and gene expression (log2 fold change ≥|2|) in *DIP2C*$^{-/-}$ #1-1 compared to RKO.

	Gene	DNA methylation Beta diff	Log2 fold change in expression
Hypomethylated	CD44	-0.51	3.42
	CDKN2A	-0.58	4.19
	FBLN5	-0.31	3.55
	FHL2	-0.34	2.11
	GGT1	-0.30	2.24
	GRPR	-0.37	-6.77
	LAMB3	-0.51	3.91
	LGALS3BP	-0.44	2.11
	PDE4DIP	-0.37	4.71
	S100A16	-0.44	2.50
	SERPINE1	-0.31	2.60
Hypermethylated	ADAMTSL1	0.69	-5.55
	CDC42BPG	0.41	-4.36
	EPS8L2	0.31	-3.73
	FLVCR2	0.33	-2.24
	GADD45G	0.36	-2.35
	IRS1	0.73	-3.57
	KCTD12	0.30	-5.33
	KRT80	0.74	-6.41
	LGI2	0.39	-4.39
	NUAK2	0.36	-2.25
	RHOBTB1	0.32	-4.10
	ROR1	0.58	-4.86
	RTKN2	0.43	-2.21
	SLC1A3	0.44	-6.66
	SLC9A2	0.34	-4.37
	TJP3	0.31	-2.25
	TMEM106A	0.33	-2.89
